# Supplementary material for: Dual-functional peptide with defective interfering genes effectively protects mice against avian and seasonal influenza
Source: Nat Commun. 2018 Jun 15;9:2358. doi: 10.1038/s41467-018-04792-7 (PMC6004018; doi:10.1038/s41467-018-04792-7)
Supplement: Supplementary file 1 — Supplementary Information [file 41467_2018_4792_MOESM1_ESM.pdf]

Dual-functional peptide with defective interfering genes effectively protects mice against avian and seasonal influenza

Zhao et al.

Supplementary information

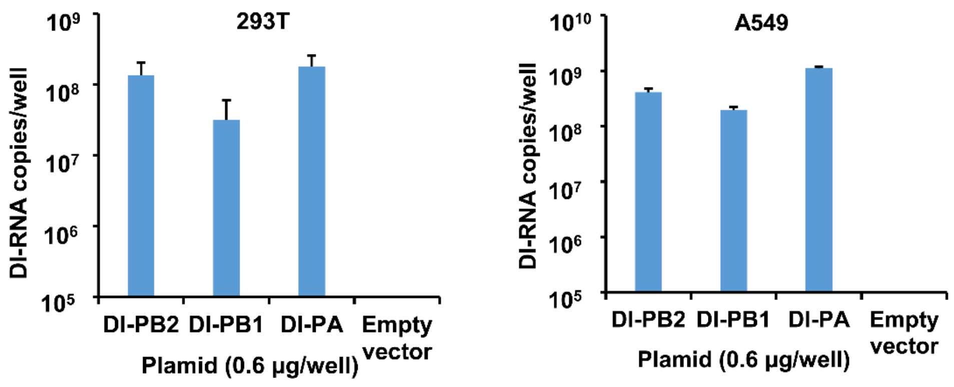

Supplementary Figure 1. Defective interfering gene expression in 293T and A549 cells when transfected individually. Plasmids of DI-PB2, DI-PB1, and DI-PA were transfected individually to cells. RNA was extracted from cells at 24 h post infection. Extracted RNA was digested by DNase I and then RNA expression was determined by RT-qPCR. Empty vector of phw2000 was used as the negative control. Data were presented as mean ± SD of three independent experiments.

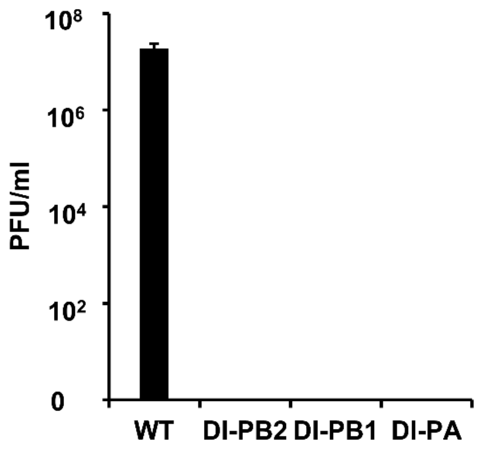

Supplementary Figure 2. Defective interfering virus did not form plaque. Viral titers of reassortant wild-type (WT: PB2-PB1-PA), DI-PB2 (DI-PB2-PB1-PA), DI-PB1 (PB2-DI-PB1-PA) or DI-PA (PB2-PB1-DI-PA) virus in the supernatants of 293T/MDCK cells. The 293T/MDCK cells were transfected with eight plasmids including eight wild-type viral genes or seven wild-type genes with one of DI-PB2, DI-PB1 or DI-PA. After 72 h post transfection, the viral titers in cell supernatants were determined by plaque assay. Data were presented as mean ±SD of three independent experiments.

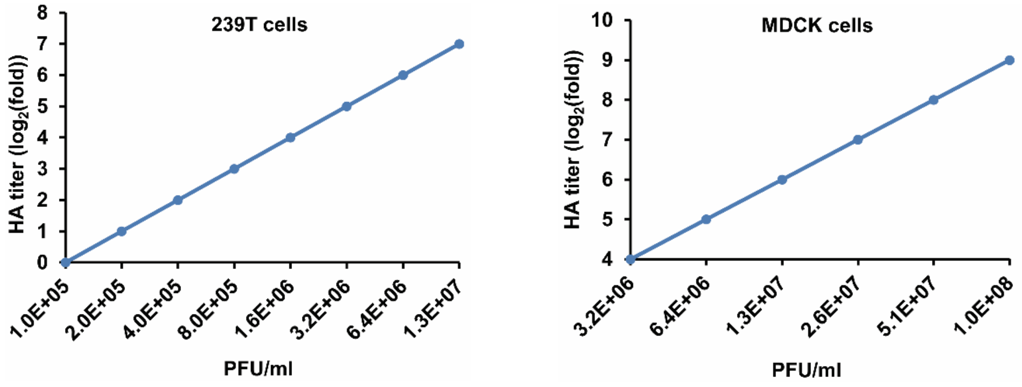

Supplementary Figure 3. Standard curve of HA titers to PFU titers of H7N7 virus grown in 293T and MDCK cells. Virus (1.3 x 10^7 PFU ml^{-1}) grown in 293T cells and virus (1.0 x 10^8 PFU ml^{-1}) grown in MDCK cells were 2-fold diluted in PBS. HA titers of each diluted virus were determined by 0.5% turkey red blood cell. Data were the average of triplicate results.

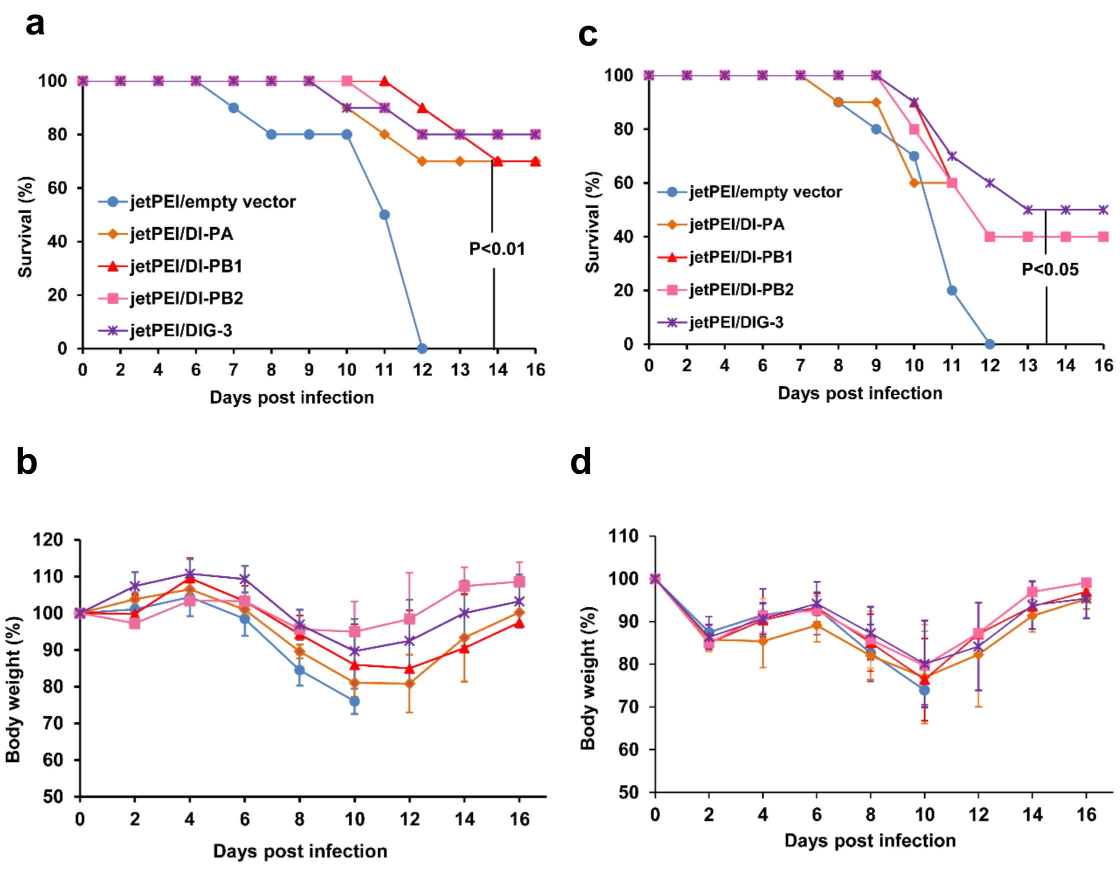

Supplementary Figure 4. Protective efficacy of DIG-3 or single DIG on mice infected by A(H7N7) virus. (**a, b**) Prophylactic efficacy of jetPEI/DIG-3, jetPEI/DI-PA, jetPEI/DI-PB1 or jetPEI/PB2 against A(H7N7) virus. (**c, d**) Therapeutic efficacy of jetPEI/DIG-3 or single DIG against A(H7N7) virus. For prophylactic experiment, 40 µl of jetPEI/empty vector (0.7 µl/ 5.0 µg in 5% glucose solution), jetPEI/DI-PA (0.7 µl/ 5.0 µg in 5% glucose solution), jetPEI/DI-PB1, jetPEI/DI-PB2, and jetPEI/DIG-3 were intratracheally inoculated to corresponding mice at 48 h and 24 h before viral inoculation. For therapeutic experiment, jetPEI/empty vector, jetPEI/DI-PA, jetPEI/DI-PB, jetPEI/DI-PB2, and jetPEI/DIG-3 were intratracheally inoculated to corresponding mice at 6 h and 24 h after viral inoculation. Survivals and body weight data were generated from 10 mice in each group with mean ± SD. *P* values were calculated by Gehan-Breslow-Wilcoxon test.

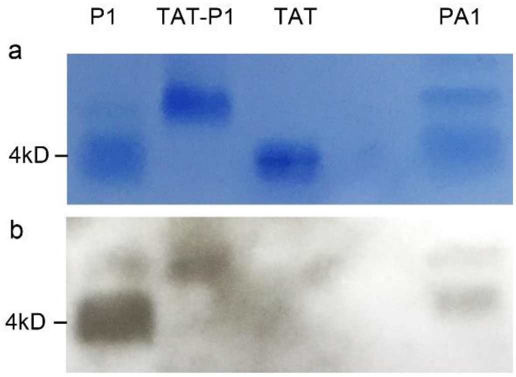

Supplementary Figure 5. Peptide binding to HA1 was determined by Western blot assay. (**a**) Peptide sizes were shown in PAGE gel stained by coomassie brilliant blue. (**b**) Binding between peptides and HA1 was confirmed by Western blot assay. One μg peptides of each was parallelly loaded to two PAGE gels. One gel was for Coomassie brilliant blue staining. The other gel was used to do Western blot assay. The peptide-transferred membrane was incubated with HA1 (2 μg ml^{-1}) and then the binding of HA1 to peptides was determined by rabbit IgG anti-HA and goat anti-rabbit IgG-HRP.

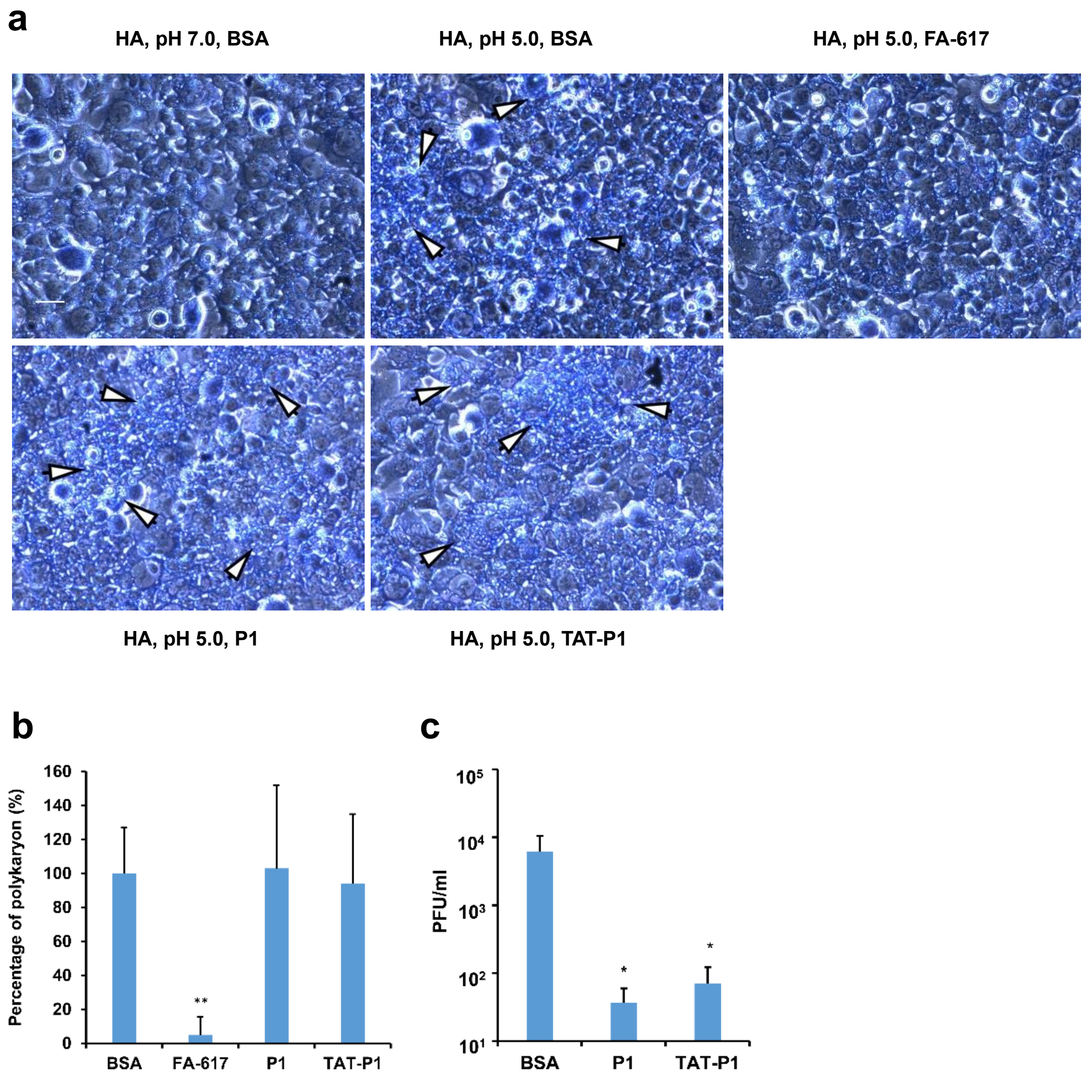

Supplementary Figure 6. P1 and TAT-P1 did not inhibit HA mediated membrane fusion. (**a**) Polykaryon formation assay. 293T cells were transfected with pH7-HA. At 24h post transfection, cells were treated with BSA (50 μg ml^{-1}), P1 (50 μg ml^{-1}), FA-617 (50 μM) or TAT-P1 (10 μg ml^{-1}) and then were further treated with pH 5.0 culture media containing the drugs. Representative images were taken after cells were cultured for 3 hours. Polykaryons are indicated by black-white arrowheads (scale bar = 30 μm). (**b**) The percentages of syncytium formation related to the control cells treated by HA-pH 5.0 were calculated from 10 microscope fields. (**c**) P1 and TAT-P1 could significantly inhibit viral replication in 293T cells. H7N7 virus (100 PFU) was pretreated with BSA (50 μg ml^{-1}), P1 (50 μg ml^{-1}l), TAT-P1 (10 μg ml^{-1}) and then was inoculated to 293T cells for culture at 37 °C. At 24 h post infection, cell supernatants were collected to determine the viral titers by plaque assay from three independent experiments. Data were present as mean ±SD of three independent experiments. * Indicates $P < 0.05$. ** Indicates $P < 0.01$ when compared with BSA. P values were calculated by the two-tailed Student's t test.

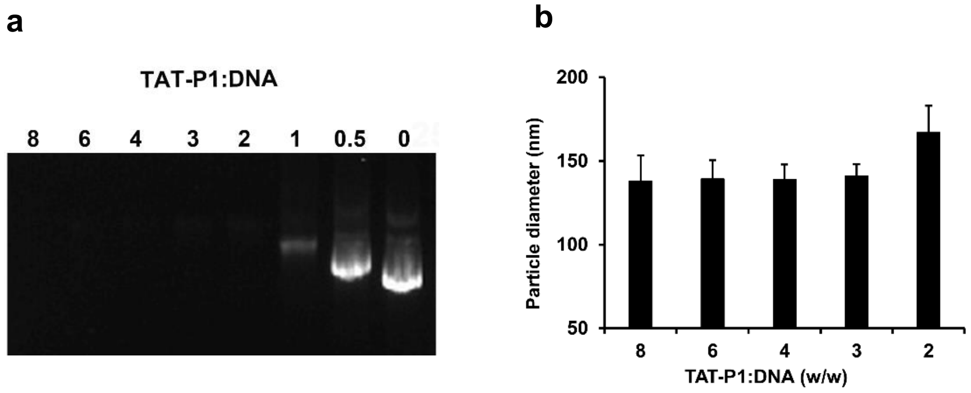

Supplementary Figure 7. TAT-P1 could bind DNA and form TAT-P1/DNA particles for transfection. (**a**) The ability of TAT-P1 binding DNA determined by gel retardation assay. (**b**) Particle diameter of TAT-P1/DNA was measured by DynaPro Plate Reader. The weight ratios of TAT-P1:DNA were indicated. Data were present as mean ±SD of three independent experiments.

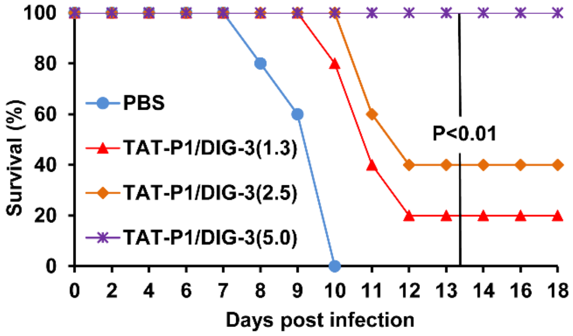

Supplementary Figure 8. The antiviral efficacy of TAT-P1/DIG-3 against A(H7N7) virus in mice was dose dependent. TAT-P1 and DIG-3 (20 μg and 5 μg) were premixed to form TAT-P1/DIG-3 complex and incubated at room temperature for 15 min. At 48 h and 24 h before viral inoculation, TAT-P1/DIG-3 including 5.0, 2.5 and 1.3 μg of DIG-3 was inoculated to corresponding mice. Survival data were generated from five mice in each group. *P* values were calculated by Gehan-Breslow-Wilcoxon test.

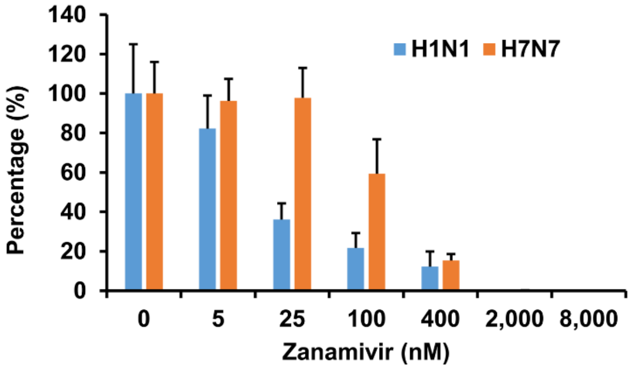

Supplementary Figure 9. A(H7N7) virus exhibited a reduced susceptibility to zanamivir when compared with A(H1N1)pdm09 virus. MDCK cells were infected with A(H7N7) and A(H1N1)pdm09 virus at an MOI of 0.005 in the presence of 0 ~ 8,000 nM of zanamivir. Supernatants were harvested at 24 h post infection and viral titers were determined by plaque assay in MDCK cells. The percentages of virus titers were normalized to the titers of virus without zanamivr treatment. Data were presented as mean ± SD of three experiments.

Supplementary Table 1. Sequence of peptides.

Peptides	Peptide sequence
TAT-P1	YGRKKRRQRRRCWGPCPTAFRQIGNCGRFRVRCCRIR
TAT-P2	YGRKKRRQRRRCWRPCPRAFRKRNCGRFRIRCCRIR
TAT-P3	YGRKKRRQRRRCWRPCPSFRQLCGRFRIRCRIR
P1	CWGPCPTAFRQIGNCGRFRVRCCRIR
TAT	YGRKKRRQRRR
P9	NGAICWGPCPTAFRQIG NCGHFKVRCCKIR
PA1	NGAICWGPCPTAFRQIG NCGHFKVRCCKIRDED

Note: TAT is from HIV-1 Tat. P1, P2, and P3 are derived from antiviral peptide P9[1]. These peptides were designed through secondary structure analysis by PredictProtein.

Supplementary Table 2. IC_{50}, CC_{50} and selective index of peptides

Peptides	MDCK					293T
	IC_{50} (H7N7) (μg ml^{-1})	IC_{50} (H1N1) (μg ml^{-1})	CC_{50} (μg ml^{-1})	Selective index (H7)	Selective index (H1)	CC_{50} (μg ml^{-1})
TAT-P1	0.56	0.67	300	535	447	226
TAT-P2	0.97	0.76	160	164	210	154
TAT-P3	0.78	0.68	207	265	304	205
P1	1.56	1.60	>400			>400
TAT	>50.0	>50.0	>400			>400

Note: IC_{50}s of peptides against A(H7N7) and A(H1N1)pdm09 viruses were determined from three independent experiments by plaque reduction assay. CC_{50}s of peptides were detected in MDCK and 239T cells through three independent experiments.

Supplementary Table 3 Oligonucleotides used for plasmid constructions

Plasmid	Primer	Oligonucleotide sequence (5' to3') [a]	Restriction enzyme
pDI-PB2	PB2-F	TATTGGTCTCAGGGAGCGAAAGCAGGTC	*Bsa*I
	PB2-MR	GGTGATACCATCACTCGGTCTG	
	PB2-MF	CGCCGGATCAGACCGAGTGATGGTATCACCTAACT GAAGACCCAGATGAAGGC	
	PB2-R	ATATGGTCTCGTATTAGTAGAAACAAGGTCGTTT	*Bsa*I
pDI-PB1	PB1-F	TATTCGTCTCAGGGAGCAAAAGCAGGCA	*Bsm*BI
	PB1-MR	ATCTGTTTGGGCATAACCAC	
	PB1-MF	ACAATGAACCAAGTGGTTATGCCCAAACAGATCAA AAGAAATCGATCCATCTTGA	
	PB1-R	ATATCGTCTCGTATTAGTAGAAACAAGGCATTT	*Bsm*BI
pDI-PA	PA-F	TATTCGTCTCAGGGAGCAAAAGCAGGTAC	*Bsm*BI
	PA-MR	TCATACAAATCTGGTAGAAACTTTG	
	PA-MF	AGAAACCAAAGTTTCTACCAGATTTGTATGAGAGAG TCCCCCAAAGGAGTGGA	
	PA-R	ATATCGTCTCGTATTAGTAGAAACAAGGTACTT	*Bsm*BI

[a]Cutting sites for restriction enzyme are underlined.

Supplementary Table 4 Sequence of DIG

Gene	Oligonucleotide sequence (5' to3')
DI-*PA*	AGCAAAAGCAGGTACTGATTCAAAATGGAAGATTTTGTGCGACAATGCTTCAATCCGAT<br>GATTGTCGAGCTTGCGGAAAAGGCAATGAAAGAGTATGGAGAGGACCTGAAAATCGA<br>AACAAACAAATTTGCAGCAATATGCACTCACTTGGAAGTGTGCTTCATGTATTCAGATTT<br>TCACTTCATCGATGAGCAAGGCGAGTCAATAGTCGTAGAACTTGGCGATCCAAATGCAC<br>TTTTGAAGCACAGATTTGAAATAATCGAGGGAAGAGATCGCACAATAGCCTGGACAGT<br>AATAAACAGTATTTGCAACACTACAGGGGCTGAGAAACCAAAGTTTCTACCAGATTTGT<br>ATGAGAGAGTCCCCCAAAGGAGTGGAGGAAGGTTCCATTGGGAAGGTCTGCAGAACT<br>TTATTGGCAAAGTCGGTATTCAACAGCTTGTATGCATCTCCACAACTGGAAGGATTTTCA<br>GCTGAATCAAGAAAACTGCTTCTTATCGTTCAGGCTCTTAGGGACAACCTGGAACCTGG<br>GACCTTTGATCTTGGGGGGCTATATGAAGCAATTGAGGAGTGCCTGATTAATGATCCCT<br>GGGTTTTGCTTAATGCTTCTTGGTTCAACTCCTTCCTCACACATGCATTGAGATAGTTGT<br>GGCAATGCTACTATTTGCTATCCATACTGTCCAAAAAAGTACCTTGTTTCTACT
DI-PB1	AGCAAAAGCAGGCAAACCATTTGAATGGATGTCAATCCGACTTTACTTTTCTTAAAAGT<br>GCCAGCACAAAATGCTATAAGCACAACTTTCCCTTATACTGGAGACCCTCCTTACAGCCA<br>TGGGACAGGAACAGGATACACCATGGATACTGTCAACAGGACACATCAGTACTCAGAA<br>AGGGGAAGATGGACAACAAACACCGAAACTGGAGCACCGCAACTCAACCCGATTGAT<br>GGGCCACTGCCAGAAGACAATGAACCAAGTGGTTATGCCCAAACAGATCAAAAGAAAT<br>CGATCCATCTTGAATACAAGCCAAAGAGGAATACTTGAAGATGAACAAATGTACCAAAA<br>GTGCTGCAACTTATTTGAAAAATTCTTCCCCAGCAGTTCATACAGAAGACCAGTCGGGA<br>TATCCAGTATGGTGGAGGCTATGGTTTCCAGAGCCCGAATTGATGCACGAATTGATTTCG<br>AATCTGGAAGGATAAAGAAAGAGGAGTTCACTGAGATCATGAAGATCTGTTCCACCATT<br>GAAGAGCTCAGACGGCAAAAATAGTGAATTTAGCTTGTCCTTCATGAAAAAATGCCTTG<br>TTTCTACT
DI-PB2	AGCGAAAGCAGGTCAATTATATTCAATATGGAAAGAATAAAAGAACTAAGGAATCTAAT<br>GTCGCAGTCTCGCACTCGCGAGATACTCACAAAAACCACCGTGGACCATATGGCCATAA<br>TCAAGAAGTACACATCAGGAAGACAGGAGAAGAACCCAGCACTTAGGATGAAATGGAT<br>GATGGCAATGAAATATCCAATTACAGCAGACAAGAGGATAACGGAAATGATTCCTGAGA<br>GAAATGAGCAGGGACAAACTTTATGGAGTAAAATGAATGACGCCGGATCAGACCGAGT<br>GATGGTATCACCTAACTGAAGACCCAGATGAAGGCACAGCTGGAGTTGAGTCCGCAGT<br>TCTGAGAGGATTCCTCATTCTGGGCAAAGAAGACAGGAGATATGGACCAGCATTAAGC<br>ATAAATGAACTGAGCAACCTTGCGAAAGGAGAGAAGGCTAATGTGCTAATTGGGCAAG<br>GAGACGTGGTGTTGGTAATGAAACGGAAACGGAACTCTAGCATACTTACTGACAGCCA<br>GACAGCGACCAAAAGAATTCGGATGGCCATCAATTAGTGTCGAATAGTTTAAAAACGAC<br>CTTGTTTCTACT

Supplementary Table 5 Oligonucleotides used for qPCR

Gene	Primer	Oligonucleotide sequence (5' to3')
H7N7-*PA*	PA-F	AACTAATCTGTATGGATTCATC
	PA-R	ATCTCGATAACGCAGTACTT
H7N7-PB1	PB1-F	ATGGCTCTTCAGCTATTCATC
	PB1-R	ATCTGATACCAACAGTCCTGC
H7N7-PB2	PB2-F	AAACTGGGAAACTGTGAAG
	PB2-R	ATCTGCTGGAATAGCGTC
DI-PA	DI-PA-F	TATTTGCAACACTACAGGGGCTG
	DI-PA-R	ATGCATACAAGCTGTTGAATACCG
DI-PB1	DI-PB1-F	CACCGAAACTGGAGCACCGCAAC
	DI-PB1-R	TTTGTTCATCTTCAAGTATTCCT
DI-PB2	DI-PB2-F	GATAACGGAAATGATTCCT
	DI-PB2-R	TCAGAACTGCGGACTCAAC
β-actin	Act-F	TGTGATGGTGGGAATGGGTCAGAA
	Act-R	TGTGGTGCCAGATCTTCTCCATGT

Reference:

1. Zhao, H. et al. A novel peptide with potent and broad-spectrum antiviral activities against multiple respiratory viruses. *Sci Rep* **6**, 22008 (2016).
